# *QuickStats*: Age-Adjusted Death Rates* from Dementia,^†^ by Sex, Race, and Hispanic Origin — National Vital Statistics System, United States, 2017

**DOI:** 10.15585/mmwr.mm6830a6

**Published:** 2019-08-02

**Figure:**
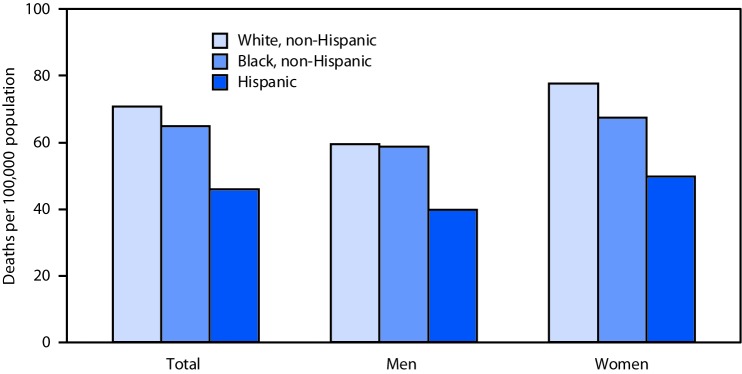
In 2017, age-adjusted death rates for dementia were higher among non-Hispanic white persons compared with non-Hispanic black and Hispanic persons (70.8 per 100,000 compared with 65.0 and 46.0, respectively). Also, among women, the rates were highest among non-Hispanic white women (77.6) compared with non-Hispanic black women (67.4) and Hispanic women (49.8). The age-adjusted death rate for non-Hispanic white men was not statistically different from the rate for non-Hispanic black men (59.4 compared with 58.8). Age-adjusted death rates were higher for women than men among non-Hispanic white, non-Hispanic black, and Hispanic populations.

**Sources:** National Vital Statistics System. Underlying cause of death, 2017. https://wonder.cdc.gov/ucd-icd10.html.

Kramarow EA, Tejada-Vera B. Dementia mortality in the United States: 2000–2017. National Vital Statistics Reports, volume 68, number 2. https://www.cdc.gov/nchs/data/nvsr/nvsr68/nvsr68_02-508.pdf.

